# Feasibility and acceptability of design and conduct of a registry-based randomised clinical trial evaluating eVIS as a digital support for physical activity in interdisciplinary pain rehabilitation programs: A randomised pilot study

**DOI:** 10.1177/20552076241299648

**Published:** 2024-11-25

**Authors:** Veronica Sjöberg, Andreas Monnier, Elena Tseli, Riccardo LoMartire, Maria Hagströmer, Mathilda Björk, Björn Äng, Linda Vixner

**Affiliations:** 1 101092 School of Health and Welfare, Dalarna University, Falun, Sweden; 2 Department of Neurobiology, Care Sciences and Society, Division of Physiotherapy, 27106 Karolinska Institutet, Huddinge, Sweden; 3 Military Academy Karlberg, 7646 Swedish Armed Forces, Solna, Sweden; 4 The Administration of Regional Board, Department of Research and Higher Education, Falun, Sweden; 5 Academic Primary Health Care Centre, 7674 Stockholm, Sweden; 6 Pain and Rehabilitation Center, and Department of Health, Medicine and Caring Sciences, 4566 Linköping University, Linköping, Sweden; 7 Biomechanics and Ergonomics Laboratory, Department of Physical Education and Sport Sciences, 37786 University of Thessaly, Trikala, Greece

**Keywords:** Chronic pain, feasibility, internal pilot study, interdisciplinary pain rehabilitation programs, patient-reported outcome measures, physical activity, randomised controlled study

## Abstract

**Background:**

Patients with chronic pain often struggle to engage in physical activity despite its health benefits. The eVISualisation of physical activity and pain intervention (eVIS) was developed to support adherence to physical activity plans in Interdisciplinary Pain Rehabilitation Programs (IPRPs) by visualising activity, pain levels, pain interference, and pharmacological use. This pilot study assesses the feasibility and acceptability of trial design and trial conduct of a registry-based randomised clinical trial (R-RCT).

**Method:**

This randomised clinical pilot study included the first 10% (n = 39, mean age 43.5, 74.4% females) of the R-RCT sample (n≈400). Participants with non-cancer chronic pain from six IPRP units were randomly assigned to either the intervention group (IPRP + eVIS, n = 19) or the control group (IPRP, n = 20). Feasibility and acceptability were evaluated using pre-defined criteria on recruitment- and data collection procedures (e.g., inclusion rates, representativeness, adverse events), physiotherapists’ ratings of trial design and conduct (e.g., acceptability, feasibility), and outcome data characteristics and completeness (e.g., adherence, data accessibility).

**Results:**

Recruitment was largely feasible, though attrition differences and the need for refined eligibility screening were noted. Physiotherapists cited time and implementation challenges. Both groups had satisfactory data completeness, but the control group showed lower adherence to daily reporting in the final third of the study. The intervention group had greater improvements in physical health, with 19.5% more participants achieving the minimum clinically important difference (≥3) on the physical component summary scale (PCS). No adverse events occurred.

**Conclusion:**

With minor adjustments, the R-RCT design is mostly feasible, though some challenges to feasibility were identified and addressed.

## Introduction

The number of people affected by chronic pain, i.e., pain experienced for more than three months or beyond the point of normal tissue healing, amounts to approximately 30% worldwide.^1,2^ The prevalence is increasing, and living with chronic pain is associated with immense physical, social, and emotional challenges for individuals as well an increased burden on societies.^3–5^ In fact, the self-rated health and health-related quality of life (HRQoL) reported by patients with chronic pain is among the lowest of any patient group.^6^

Physical activity as treatment in chronic pain is demonstrably evident and well-implemented in primary and specialist healthcare as it provides relief to emotional and physical wellbeing, enhances overall physical function, and decreases levels of pain intensity.^7–9^ Current physical activity guidelines, applicable to the general population as well as those with chronic conditions, recommends engaging in at least 150 min of moderate to vigorous intensity physical activity (MVPA) per week.^10^ Despite its established benefits, there are many reports on the inadequate levels of physical activity among those living with chronic pain.^11–14^ Examples of known barriers to reaching and maintaining beneficial levels of physical activity among individuals living with chronic pain include fear-avoidance beliefs, inaccurate health care advice, comorbidities, pain, low motivation, and a lack of time and social support.^15–19^ Clearly, there is a gap between the well-established effectiveness of physical activity as a treatment and patients’ ability to adhere to given advice and regimens. This gap may be explained by the fact that many treatments, including physical activity, require patients to actively take part in the treatment, to do so regularly, and, in some cases, to continue to do so for the long term, all of which requires them to embrace long-term behaviour change.^20^ Several theories in behavioural medicine explain the complex interactions between behaviour and health outcomes.^21^ Due to this, the development and evaluation of interventions and techniques targeting individually beneficial physical activity levels with elements of behaviour change have undergone a lot of development.^22–24^ In particular, behaviour change techniques such as goal setting, social support, incentives, self-monitoring, and behavioural practice seem to be effective in promoting healthy physical activity behaviours within various populations, including patients living with chronic pain.^24–26^

Chronic pain management adopts a biopsychosocial framework, emphasising holistic care across all dimensions, commonly integrated into Interdisciplinary Pain Rehabilitation Programs (IPRP).^27^ These programs, rooted in behavioural therapy and education principles, offer comprehensive, synchronised interventions spanning psychological, medical, social, occupational, and physical aspects, facilitated by a collaborative, multi-professional team.^28^ However, due to the diverse nature of chronic pain sufferers in terms of pain characteristics and socio-demographic factors, and the modestly superior effectiveness of IPRP compared to single treatment approaches,^29–31^ there's a growing call to individualise pain medicine and management.^20,32^

**Figure 1.**
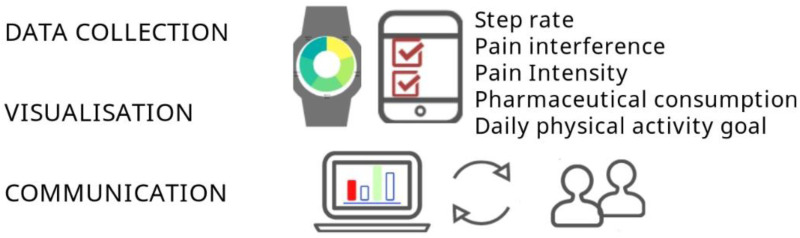
Schematic illustration of core features in eVIS; Data collection, Visualisation and Communication of patient-reported outcome measures (PROMs); pain intensity, pain interference, pharmaceutical consumption, and objectively registered physical activity (steps/day).

To address the gap between physical activity-based treatments in IPRP, patient outcomes, and adherence to advice, the eVISualisation of physical activity and pain intervention (eVIS) was developed (Figure 1). The intervention underwent continuous development and evaluation following recommended phases outlined by the updated Medical Research Council (MRC) framework for developing and evaluating complex health interventions.^33^ Initially, we evaluated the wrist-worn activity tracker's validity, demonstrating acceptable validity in measuring step rate.^34^ Subsequently, we refined and evaluated data collection, visualisation, communication features, and the intervention overall, including assessing pre-clinical content validity and feasibility within IPRPs.^35^ Moreover, as per the updated MRC framework, complex health interventions must be theoretically grounded. eVIS is theoretically anchored in Social Cognitive Theory, which offers behaviour change techniques targeting beneficial physical activity levels, including goal setting, outcome expectations, self-monitoring, education, and self-efficacy.^36–38^ The intervention aims to facilitate patients’ understanding and experience of being physically active despite pain, aligning with core concepts in Pain Neuroscience Education.^39^

Prior to evaluating intervention effectiveness, it's crucial to assess the feasibility and acceptability of trial design and conduct within the intended context, aiming to enhance implementation success, data quality, and internal validity.^40–42^ Success in evaluating effectiveness relies heavily on intervention implementation, influenced by user and deliverer acceptability—defined as stakeholders’ cognitive and emotional responses to the intervention.^43^ Evaluating study design feasibility in early stages helps identify uncertainties related to context, program theory, stakeholder perspectives, and responses to intervention, guiding trial refinement.^33,43,44^ Therefore, this study aims to assess the feasibility and acceptability of conducting a registry-based randomised clinical trial (R-RCT).

## Method

### Research design

This pilot study served as the initial phase of a two-armed pragmatic R-RCT,^40,45^ as outlined in detail in the study protocol by Sjöberg et al.^46^ Feasibility and acceptability aspects in the R-RCT's design and conduct were assessed against pre-defined criteria.^40–42^ To ensure high reporting standards, the study adhered to the Consolidated Standards of Reporting Trials (CONSORT), along with its extension for pilot trials.^47^ A full CONSORT checklist for pilot trials is available in Supplementary Material.

### Setting

This study was conducted within six small to medium-sized outpatient specialised IPRP units in Sweden (annual patient throughput 40–300). At the time of the study, there were approximately 60 units offering IPRP in Sweden, with two-thirds organised as part of specialist healthcare. Patients are usually referred to IPRP from primary care when they have complex chronic pain that significantly affects their daily lives.^48^ Prior to acceptance into rehabilitation, patients are assessed based on patient-related, care-process-related, and care-giver- related criteria, as detailed elsewhere.^49^ IPRP typically include dialogue and education, activity training, meetings, psychological treatments, relaxation, and physical exercise.^48^ The biopsychosocial nature of the approach involves synchronised actions delivered by at least two professions. There is no standardised recommendation regarding the composition of treatments, duration (varies between 1–18 weeks), or intensity (hours of contact) of IPRP.^31,50^

### Randomisation and allocation

A computer-generated randomisation design with random block sizes of 4 and 6 was used to allocate participants to either the intervention group (IPRP with an addition of eVIS, n = 19) or the control group (IPRP, n = 20). The randomisation schedule was performed by a representative of the technical company that developed PATRON (Nordforce Technology AB). Sequentially numbered opaque sealed envelopes with intact blocks were used to ensure allocation concealment. Allocation took place at the IPRP unit and was conducted by physiotherapists from the IPRP team. Neither the patients nor the IPRP team were blinded to the intervention due to its nature.

### Education module

All physiotherapists participated in a two-hour digital education module conducted by study staff, followed by at least one additional follow-up module. The education module provided comprehensive information on the study design, which was broken down into specific study activities, including informing potential participants, conducting eligibility screenings, collecting informed consent, and the allocation procedure. The education also included an in-depth presentation of the intervention, as well as information on the logical model of the intervention, including the proposed mechanisms of action. Physiotherapists were informed on how eVIS was intended to supplement IPRP, and they were strongly encouraged to integrate eVIS into their IPRP by facilitating regular shared reviews of patient data at each appointment. This approach was designed to promote discussions with patients on their reflections regarding goal achievement, trends, and variations within and between the separate variables.

### Intervention group

Participants in the intervention group took part in the unit's standard IPRP programme with the addition of eVIS for a coherent time span of six months, including the IPRP time. The addition of eVIS implied using the wrist-worn activity tracker and to record daily pain intensity, pain interference, and pharmaceutical consumption in PATRON. Physiotherapists at the unit were encouraged to prompt shared viewing of patient data in the intervention visualisation feature at each appointment.

### Control group

Participants in the control group followed the standard IPRP program offered at the unit and were asked to record their data in PATRON, just like the intervention group, over the six-month study period. However, they did not use a wrist-worn activity tracker or have access to PATRON's visualisation interface.

### Data collection and outcomes

Data on feasibility and acceptability were collected from several sources, informing on three main feasibility areas: 1. Recruitment and data collection procedures, including the occurrence of adverse events, 2. Physiotherapists’ ratings on the feasibility of the R-RCT study design and the acceptability of the trial conduct and the intervention, and 3. Characteristics and completeness of primary and secondary outcome data.

### Feasibility and acceptability of recruitment and data collection procedures

#### IPRP units and staff

Recruitment of IPPR units and IPRP staff was initiated in the late fall of 2021 by distributing information flyers to approximately 50 of the approximately 60 IPRP units in Sweden (including both primary and specialised units). The flyer included information about the study and an invitation to attend an open digital information meeting. IPRP units were selected for the study according to the following criteria:

- Affiliation with the Swedish Quality Registry for Pain Rehabilitation
- Patient throughput >20 per year
- Adherence to national criteria for IPRP^49^
- Approval for participation provided by unit management

Units that participated in one of the digital information meetings were contacted by email to follow-up on their interest in participation. If participation was accepted, written consent was collected from management and a two-hour digital training module was scheduled. The training module covered overall information on the study design, rationale for the intervention, and detailed information and instructions for study procedures. In addition, access to a designated website was provided, which contained essential information, documentation forms, and step-by-step guides for study activities. In order to maintain the pragmatic nature of the study design, only general information on the main ideas of how eVIS would be applied in IPRP was presented, plus suggestions of verbal prompts (reflective questions) to facilitate discussion between patients and physiotherapists based on eVIS data.

#### Patients

Patients offered a place in this study had been accepted into IPRP due to non-malignant chronic (>3 months) musculoskeletal or general widespread pain. They were aged between 18 and 67 years old, able to comprehend written and verbal instructions in Swedish, and had daily access to an internet browser on a smartphone, computer, or tablet. Patients requiring walking aids were considered ineligible. Physiotherapists in the IPRP teams provided both written and verbal information about the study and performed eligibility screenings, carefully documenting the outcomes. Written informed consent was obtained from each participant prior to study start. Participants in this pilot study comprise the first 10% who completed the study period in the R-RCT. The recruitment process is described in detail elsewhere.^46^

### Physiotherapists’ ratings on feasibility and acceptability of trial design and trial conduct

All physiotherapists at the participating IPTP units received a purpose-developed questionnaire on a single occasion. The questionnaire was distributed via email through Sunet Survey in November 2022 after being piloted within parts of the author group. The questionnaire comprised 31 items that covered five main areas of feasibility: 1. Willingness/attractiveness to participate as an IPRP unit in the study, 2. Acceptability of performing study activities, 3. Feasibility of current study conduct, 4. Implementation process, and 5. Adverse events related to eVIS. Of the total 31 items, 26 used a 4-point Likert scale with the following explanations of grades 1: Not at all, 2: To some extent, 3: To a rather large extent, 4: To a large extent. Of the remaining five items, one item used a 5-point Likert scale with the following explanations of grades: 1. 0%, 2. 1–24%, 3. 25–49%, 4. 50–74%, 5. 75–100% (“To what extent (in relation to the total number of times you have met or been in contact with the patient) would you say that, on average, you have used eVIS as a complement to the patient's MMR?”). Two items provided for a free text response (“Overall, during the time you have participated in the study, what has facilitated and/or hindered your participation in the study?” and “Here you can provide any other information you believe may be of interest to us), one item had a dichotomized (yes/no) response (“Indicate here if you have experienced (personally or through information from patients) any unexpected events with eVIS”), and one had a response scale of 0–10 where 0 meant ‘not at all appropriate’ and 10 meant ‘very appropriate’ (“To what extent do you consider eVIS to be appropriate as a supplementary treatment to IPRP”?). Respondents were encouraged to provide free text comments for all items.

### Feasibility of the characteristics and completeness of outcome data

#### Primary outcome variable in R-RCT

The primary outcome variable in the forthcoming R-RCT is the Physical Component Summary Scale (PCS), which ranges from 0–100 points with high values indicating better physical health. PCS is a calculated measure derived from the health survey RAND-36 (equivalent to SF-36, version 1).^51,52^ In this pilot study PCS was calculated according to Taft et al. (2001), using norm values from the Swedish population provided from Swedish Quality Registry for Pain Rehabilitation.^53^ The measurement properties of PCS have been evaluated for individuals living with chronic pain, supporting acceptable structural validity, test-retest reliability, and responsiveness to identify changes over time.^54–56^ At baseline and at the six-month follow-up after IPRP, all participants completed the RAND-36 digitally. In order to minimize data loss, up to four reminders were distributed via e-mail and SMS.

In order to interpret clinically relevant changes in PCS and establish the effectiveness of eVIS, a Minimal Clinically Important Difference (MCID) was applied.^55^ In this pilot study, we adhere to the well-accepted definition of an MCID as the smallest change in a patient's PROM that constitutes a beneficial difference between two measurement points, without extreme costs or burdensome side effects.^56^ Several MCIDs for PCS have been proposed, varying from 2.06 to 5.73, however, an MCID of 3 is commonly applied for PCS.^31,57–60^

#### Secondary outcome variables in R-RCT

Daily PROMs for pain intensity and pain interference with daily activities were recorded. Pain intensity was assessed using a numeric rating scale, where participants were asked to record the number corresponding to their average pain over the last day with 0 indicating no pain at all and 10 indicating the worst imaginable pain.^61,62^ Pain interference with daily activities was evaluated using a numeric rating scale where participants indicated to what extent their daily activities were affected by their pain over the last day, with 0 indicating not at all and 10 indicating to a very large extent.^35^ These two assessments were both registered in the web application and participants could not choose to only answer one. In addition, participants in both the intervention and control groups were instructed to register their daily pharmaceutical consumption (name, form, dose, strength) in the web application. For participants in the intervention group, daily data on objectively measured physical activity (step rate) was collected using the wrist-worn activity tracker (Fitbit Versa 2) and synchronised with the web application. The reliability of the activity tracker's step measurements has been evaluated and deemed acceptable for this patient group.^34^

### General feedback from participating patients

In addition to patient-reported quantitative data, voluntary free-text feedback was collected from participants in both groups by adding one extra item to the RAND-36 questionnaire at the six months follow-up. The question was phrased as follows: “Please feel free to provide any type of feedback on matters you have found relevant during the study period”. These free-text comments were categorised descriptively into themes at a manifest level.

### Adverse events related to trial design or trial conduct

Aside the item in the physiotherapist questionnaire on adverse events, all participating IPRP staff including managers, and participating patients were strongly and repeatedly encouraged to report any adverse events (such as symptom deterioration or dermatological issues associated with the use of the wrist-worn activity tracker) that may be related to eVIS or the implementation of eVIS within IPRP. Events could be reported by email or telephone.

### Statistical analyses and feasibility criteria

All calculations were performed in SPSS, version 26. In order to evaluate the feasibility of study design and study conduct, per protocol analyses were performed. Data on recruitment- and data collection procedures, the characteristics of included IPRP units and patients were presented descriptively. Physiotherapists’ ratings of feasibility on four-point Likert scales were calculated and presented as frequencies and percentages. Items with a 0–10 response scale were presented as median and interquartile range (IQR). Characteristics (mean, SD) of the primary outcome in the R-RCT were descriptively calculated and presented both as totals and for each group to enable the assessment of the sample compared to the goal population. In addition, differences in percentages, including 95% confidence intervals of patients who improved from baseline to follow-up by MCID of ≥3 points were calculated to provide data on the primary outcome to enable a final sample size calculation

In order to assess the completeness of secondary outcome data in the R-RCT, the adherence to daily registrations was calculated. First, we calculated participants number of *valid weeks*. A valid week was defined as one in which a participant completed at least four of the seven possible registrations, thereby providing sufficient data for visualisation in the web application. This visualisation enabled the monitoring of trends and variations in the data. Secondly, we established an acceptable threshold for the total number of valid weeks over the study period, which was set at ≥70%, or at least 18 out of 26 weeks. Frequencies and proportions of participants who met the acceptable level of valid weeks were calculated for each group and overall. In order to assess participants’ adherence to daily registrations over the study period, frequencies and proportions of participants who met the acceptable level of valid weeks were calculated for the first part of the study period (weeks 1–8), the middle part (weeks 9–17), and the last part (weeks 18–26). In accordance with methodological recommendations for testing R-RCT feasibility, several feasibility criteria were defined.^41,42^

**Satisfactory recruitment and data collection procedures**

–  ≥ 50% of planned IPRP units (n = 15) were recruited within the first six months.
– Included IPRP units were representative of Swedish IPRP in terms of professions, programme duration, and programme intensity.
– Included patients were representative of the target population regarding personal and pain characteristics.
– Complete baseline and follow-up data for ≥10% of the goal population within the first six months.
–  < 1 adverse event occurring.

**Satisfactory feasibility and acceptability of trial design and trial conduct rated by physiotherapists in IPRP**

– Satisfactory feasibility (≥3) was rated by physiotherapists on the study activities designed to attract IPRP units.
– Satisfactory feasibility (≥3) was rated by physiotherapists on their ability to perform study activities and their willingness to randomly allocate participants.
– Satisfactory acceptability (≥3) was rated by physiotherapists on the conduct of study activities.
– Satisfactory feasibility and acceptability (≥3) were rated by physiotherapists on the implementation process.

**Satisfactory feasibility of outcome data's characteristics and completeness**

–  < 20% missing in primary outcome data due to dropout or failed completion of baseline or follow-up data collection.
– For each secondary outcome data, data completeness was assessed as satisfactory if ≥70% of participants provided valid data (i.e., ≥ 4 of 7 registrations per week) for at least 18 weeks of the study period (26 weeks).
– Acceptable longitudinal adherence to data collection, i.e., ≥ 70% provided valid data in each study period.
– Satisfactory ratings of data accessibility by involved researchers.

An overall assessment of the above outlined criteria was performed in order to identify potential issues in the design and conduct of the R-RCT. Assessments were categorised as s*atisfactory* (no changes in design or conduct required), *moderately satisfactory* (changes in design or conduct may be required), or *insufficient* (the feasibility of the design or conduct is not appropriate).^41^

## Results

### Feasibility and acceptability of recruitment and data collection procedures

Of the approximately 50 units invited to information meetings, 23 attended. Over the course of six months, seven units chose to participate in the study. Unfortunately, one unit decided to drop out almost immediately due to staffing difficulties. The patient volume per year among participating units ranged from 40 to 300 under normal circumstances (Table 1). However, all units reported a reduced patient volume due to the Covid-19 pandemic, resulting in approximately halved patient volumes during the study period. The number of professions represented at the IPRP units ranged from three to six. Programme duration ranged from four to 20 weeks, and the programme intensity varied from full-time participation to one day per week. All of the units adhered to the national criteria for IPRP^49^ and reported PROM data to Swedish Quality Registry for Pain Rehabilitation.

**Table 1.** Characteristics of participating IPRP units.

IPRP units	Pat./year	Professions represented in IPRP teams	Main treatment form	Programme duration	Programme intensity
1	∼120	Physiotherapist, physician, occupational therapist, access to behavioural scientist and psychologist (n = 3/n = 5)	Group based (only)	4 weeks	6–8 h/day
2	∼100	Physiotherapist, physician, psychologist social worker, rehabilitation coordinator (n = 5)	Group based (mostly)	8–12 weeks	2-3 days/week for 8 weeks or 1 day/ week for 4 weeks
3	∼300	Physiotherapist, physician, psychologist, nurse with psychotherapist competence, access to occupational therapist (n = 4/n = 5)	Individual (only)	12–20 weeks	3-4 days/week 1-1.5 h/day
4	∼80	Physiotherapist, physician, occupational therapist, psychologist, nurse (n = 5)	Individual (only)	Tailored to patient´s needs	Maximum 2 h/day
5	∼40	Physiotherapist, physician, occupational therapist, psychologist, social worker, nurse (n = 6)	Individual and group based	13/15 weeks	3 days/week 4 h/day
6	∼80	Physiotherapist, physician, occupational therapist, psychologist (n = 4)	Group based (mostly)	5 weeks	3 days/week 7 h/day

**Figure 2.**
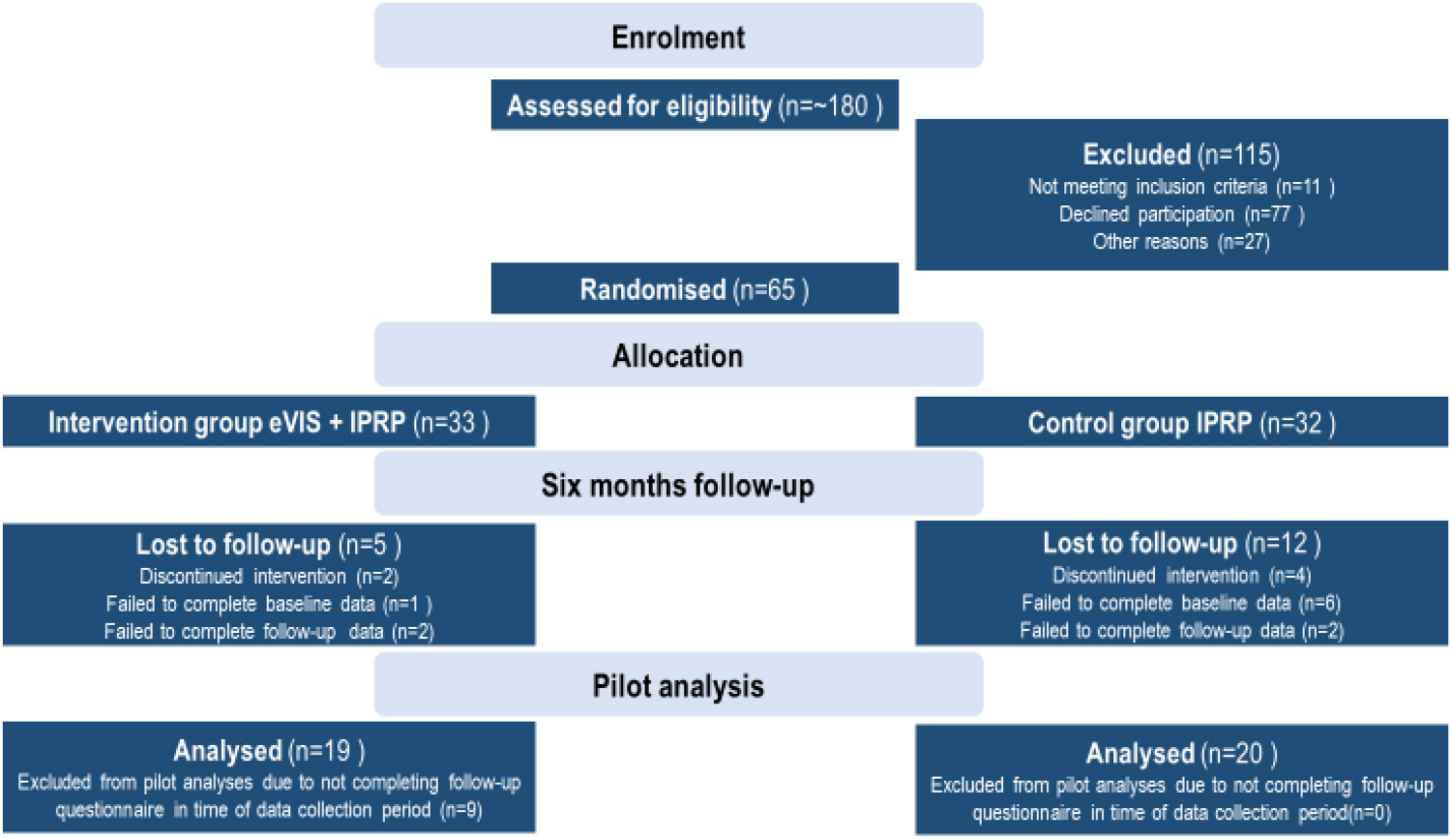
Consolidated standards of reporting trials (CONSORT) flow chart of study design.

Between November 2021 and December 2022, ten physiotherapists at seven IPRP units assessed around 180 patients for eligibility, randomly assigning 65 patients into two groups – 33 into the intervention group and 32 into the control group. Among the remaining 115 patients, the majority declined participation (n = 77), often citing concerns about adding extra stress to their rehabilitation program. Eleven patients didn’t meet inclusion criteria, and 27 were excluded for unknown reasons. Of the randomised patients, 17 were lost to follow-up due to drop-out, incomplete baseline- or follow-up questionnaires resulting in a 15% attrition rate in the intervention group (5 out of 33) and 37.5% in the control group (12 out of 32). Refer to Figure 2 for a Consolidated Standards of Reporting Trials (CONSORT) flow chart outlining the study design and allocation procedure.

Patient inclusion for the R-RCT and pilot study began in late 2021 and continued until at least 15 participants in each group provided baseline and follow-up data. Table 2 outlines participants’ personal and pain characteristics. The mean age was approximately 44 years (SD 11.4), with 74% being female. In the intervention group, the mean age was 45 years (SD 12), with 90% females. Conversely, the control group had a mean age of 42 years (SD 10), with 60% females.

**Table 2.** Overview of personal and pain characteristics for participating patients, presented as a total and per group.

	Total (n = 39)	Intervention group (n = 19)	Control group (n = 20)
**Personal and pain characteristics**			
Age years, mean (SD)	43.5 (11.4)	45.3 (12.4)	41.8 (10.3)
Sex, % female	74.4	89.5	60.0
PROM pain intensity (0–10), median (IQR)	6.0 (4)	6.0 (3]	7.0 (4)
PROM pain interference (0–10), median [IQR]	5.0 (3)	5.0 (3)	5.0 (5)
Pharmaceutical consumption, percent (n)	100 (39)	100 (19)	100 (20)
Step rate, median [IQR]	N/A	7894 [5500]	N/A

Abbreviations: SD = Standard deviation, PROM = Patient-reported outcome measure, IQR = Interquartile range.

Eligibility screening documentation revealed that one unit did not consistently document procedures for ineligible or declining patients. Physiotherapists rarely required additional support during the study, but approximately 12–15 patients needed personal assistance, mainly due to issues like activation code failures for PATRON, data synchronisation problems with the activity tracker, and difficulty pairing it with the Fitbit app. Information material was revised accordingly as support needs arose. However, three participants encountered a critical error with the web application, resulting in a “white screen”. Extensive troubleshooting led to improvements in the application's code library. Researchers found accessing data on primary and secondary outcomes satisfactory, with only minor issues corrected in the data export process.

### Physiotherapists’ ratings on the feasibility and acceptability of trial design and conduct

Seven physiotherapists from five IPRP units provided ratings of feasibility and acceptability of the trial design and its concept using four-point Likert scales. In general, physiotherapists working within IPRP found eVIS to be a feasible intervention in their setting. They also deemed the study activities, including providing information, assessing eligibility, obtaining consent, allocating patients, answering questions, and assisting patients in both groups to be feasible and acceptable (Table 3).

IPRP physiotherapist unit E: We received clear instructions and well-structured documents and information material – just for us to follow. It's been easy thanks to this and the support we received via e-mail. However, the additional time it takes to conduct the study has sometimes been a challenge due the fact that we [the physiotherapists] already have full calendars.

**Table 3.** Detailed overview of frequencies and percent of physiotherapists ratings ≥**3** of feasibility and acceptability per item.

Items of feasibility and acceptability rated by physiotherapists (n = 7)	Ratings ≥3, n (%)
**Feasibility of study activities designed to attract IPRP-units**	
Digital information meeting	5 (71)
Designated web site	1 (14)
**Acceptability of study activities**	
Provide relevant information to patient	7 (100)
Assess eligibility	7 (100)
Collect informed consent	7 (100)
Perform randomisation	7 (100)
Questions related to patient’s allocation	6 (86)
Assist patient in intervention group	4 (57)
Assist patient in control group	5 (71)
**Perceived feasibility of conduct of study activities**	
Safely storage study material	7 (100)
Procedures of providing study information	4 (57)
Procedures of eligibility screening	7 (100)
Procedure of collecting consent	7 (100)
Procedure of randomisation	7 (100)
Procedure of handling patient after group allocation	5 (71)
Procedure of start-up in intervention group	4 (57)
Procedure of start-up in control group	5 (71)
**Perceived feasibility of implementation process**	
Use web application as support to physical activity	3 (43)
Procedure of incorporating eVIS in treatment	3 (43)
Time resources	1 (14)
Technical experience	5 (71)
Support from management	1 (14)
Support from colleagues not participating	3 (43)
Support from colleagues participating	5 (71)
Perceived potential of eVIS	2 (29)
Perceived patient interest to participate	1 (14)
Use of eVIS in >25% of total number of available patient appointments	1 (14)
Perceived appropriateness of eVIS (0–10)*, median [IQR]	5 [4]
Occurrence of adverse events, n	0

PT = Physiotherapist, * Response scale 0 = not appropriate at all, 10 = very appropriate, IQR = Interquartile range.

However, there were a few exceptions. Regarding the provided activities designed to attract IPRP units and staff to participate in the study, the designated website aimed at supporting the IPRP units in study activities was rated as feasible by only one physiotherapist. A few areas related to the implementation of the R-RCT study design in IPRP were identified as problematic by the other physiotherapists. These included potential barriers such as insufficient time resources and support from management. In addition, some physiotherapists did not perceive patients as being interested in using eVIS, as only one out of seven physiotherapists rated such patient interest as feasible.

*IPRP physiotherapist unit A: I have received full support [from management], but no additional resources.*

*IPRP physiotherapist unit F: For some people, eVIS may increase motivation. For others, it is seen as a stressful tool.*

*IPRP physiotherapist unit D: Many [patients] decline participation due to IPRP containing too many tasks.*

*IPRP physiotherapist unit A: Many [patients] want to focus on their IPRP without extra additions, and some patients believe that six months [the length of the study period] is far too long.*

Physiotherapists’ evaluations of eVIS's perceived potential to improve pain management and patient's interest in participating in the study both received low ratings. Only two of seven physiotherapists thought that eVIS had a high potential to contribute to pain management. Only one physiotherapist rated patient interest as high. Some free-text comments from both physiotherapists and patients may offer explanations, such as the perception that the rehabilitation regimen was already burdensome, and that this burden may be further increased by the addition of eVIS.

Ratings on the perceived appropriateness of eVIS use in IPRP (0–10, 0 = not at all appropriate, 10 = completely appropriate) were moderate, with a median score of 5 (IQR 4). One of the seven physiotherapists reported applying eVIS in more than 25% of their total patient appointments, while the remaining six reported using eVIS in 1–25% of their patient appointments.

### Feasibility of the characteristics and completeness of outcome data

#### Feasibility of the primary outcome in the R-RCT

In order to assess the feasibility of the primary outcome of the R-RCT, characteristics and completeness of outcome data were examined. Participants in the intervention group rated their physical health using the PCS (0–100) domain in RAND-36, with an average score of 30.40 (SD 8.05) at baseline and 38.39 (SD 11.96) at follow-up. In the control group, these scores were 31.90 (SD 6.20) at baseline and 35.13 (SD 9.36) at follow-up (Table 4). Table 5 shows the mean and standard deviation of PCS scores at both baseline and follow-up, as totals and per group.

**Table 4.** Characteristics (mean and SD) of primary outcome (Physical Component Summary Scale [PCS]) at baseline and follow-up presented per group and in total.

	Total, n = 39		Intervention group, n = 19		Control group, n = 20	
	Baseline	Follow-up	Baseline	Follow-up	Baseline	Follow up
PCS 0–100*, mean (SD)	**30.66** (7.07)	**36.72** (10.69)	**30.40** (8.05)	**38.39** (11.96)	**30.90** (6.20)	**35.13** (9.36)

* Calculated according to Taft et al.^53^

Table 5 shows the proportions of participants who showed improvement, no change, or deterioration from baseline to follow-up in PCS, using a cut-off of MCID ≥ 3. There was a 19.5% difference in the number of participants showing improvement between the two groups, In the intervention group, 17 participants (90%) were categorised as improved, compared to 14 participants (70%) in the control group.

**Table 5.** Proportions, frequencies, and 95% confidence intervals (CI) of improved, unchanged, or deteriorated patient from baseline to follow-up on the primary outcome Physical Component Summary Scale (PCS), applying an MCID of ≥3p.

	MCID ≥3 p			
	Intervention (n = 19) % (frequency)	95% CI	Control (n = 20), % (frequency)	95% CI
Improved	89.5 (17)	68.6 to 97.1	70.0 (14)	48.1 to 85.5
No change	5.3 (1)	0.9 to 24.6	15 .0 (3)	5.2 to 36.0
Deteriorated	5.3 (1)	0.9 to 24.6	15.0 (3)	5.2 to 36.0

MCID: Minimal Clinically Important Difference.

#### Feasibility of secondary outcomes in the R-RCT

In the intervention group, one participant completely avoided registering pain intensity/pain interference, however, the remaining 18 participants (95%) had at least 70% of the study period of 26 weeks as valid weeks of registrations (Table 6). When assessing long-term adherence to registrations throughout the study period, 18 participants (95%) in the intervention group consistently maintained a high adherence across all three study periods. In contrast, adherence in the control group was slightly lower. For the entire study period, 14 participants (70%) provided at least 18 valid weeks of registrations. Long-term adherence in the control group initially increased in the first study period where 18 participants (90%) achieved at least 70% valid weeks. However, this level of adherence decreased in the final study period.

**Table 6.** Overview of completeness in secondary outcome data per group and in total. Proportions and frequencies of participants who achieved ≥70% (18/26) valid weeks (one valid week = ≥ 4 of 7 completed registrations) of patient-reported and objectively measured outcome data for the entirety of the study period, and in the first (study period 1), second (study period 2), and last (study period 3) parts of the study.

	Total (n = 39)	Intervention group (n = 19)	Control group (n = 20)
**PATIENT-REPORTED OUTCOMES**			
**For the entire study period (26 weeks)**			
Participants with ≥70% valid weeks of pain intensity/pain interference* reports (0–10), % (n)	85 (33)	95 (18)	70 (14)
Participants with ≥70% valid weeks of pharmaceutical consumption report, % (n)	82 (32)	95 (18)	70 (14)
**For study period 1 (week 1–8)**			
Participants with ≥70% valid weeks of pain intensity/pain interference report (0–10), % (n)	92 (36)	95 (18)	90 (18)
Participants with ≥70% valid weeks of pharmaceutical consumption, % (n)	95 (37)	100 (19)	90 (18)
**For study period 2 (week 9–17)**			
Participants with ≥70% valid weeks* of pain intensity/pain interference report (0–10), % (n)	85 (33)	95 (18)	75 (15)
Participants with ≥70% valid weeks of pharmaceutical consumption, % (n)	87 (34)	95 (18)	80 (16)
**For study period 3 (week 18–26)**			
Participants with ≥70% valid weeks of pain intensity/pain interference report (0–10), % (n)	77 (30)	95 (18)	70 (14)
Participants with ≥70% valid weeks of pharmaceutical consumption, % (n)	74 (29)	95 (18)	65 (13)
**OBJECTIVELY MEASURED OUTCOME**			
**For the entire study period (26 weeks)**			
Participants with ≥70% valid weeks of step rate, % (n)	N/A	90 (17)	N/A
**For study period 1 (week 1–8)**			
Participants with ≥70% valid weeks of registered step rate	N/A	100 (19)	N/A
**For study period 2 (week 9–17)**			
Participants with ≥70% valid weeks of registered step rate	N/A	95 (18)	N/A
**For study period 3 (week 18–26)**			
Participants with ≥70% valid weeks of registered step rate	N/A	90 (17)	N/A

* Ratings of pain intensity and pain interference are presented jointly as the items are jointly mandatory in the web application.

All participants in both groups registered their pharmaceutical consumption. Eighteen (95%) of participants in the intervention group, and 14 (70%) in the control group met the criteria of ≥70% valid weeks for the entirety of study period (Table 6). This high adherence to registrations was overall, maintained throughout the three study periods for both groups. In particular for participants in the intervention group, where participants with valid weeks was at least 95% no matter study period. However, the adherence in the control group decreased throughout the study. From 18 (90%) in the first period, to 16 (80%) in the second, and below cut-off, 13 (65%) for the final study period.

In general, the reported consumption was distributed among three areas within the Anatomic Therapeutic Chemical Classification Systems (ATCs), more particularly within ACT C (cardiovascular system), ACT N (nervous system) and ACT M (musculoskeletal system). The most frequently reported for both groups were the ACT N02BE01 representing different brands of a common, over the counter pharmaceutical ATC, Acetaminophen (Paracetamol). Five participants (2 from intervention group, 3 from control group) reported consumption of opioids and/or psycholeptics (N02AJ06, N02AA05, N05BA04).

Seventeen participants (90%) in the intervention group provided physical activity data for at least 18 of the 26 valid weeks. Participants in this group consistently adhered to measuring physical activity at a high level over the study period, with all 19 participants reaching satisfactory levels of measurement in first study period, 18 (95%) in the second study period, and 17 (90%) in the final study period (Table 6). Regarding participants’ daily activity goals, it ranged between 5000 and 15,000 steps per day. Goal revision was rare; eight participants revised their daily activity goal once during the study period. One participant revised the goal daily (178 times), and one revised it on two occasions.

### General feedback from participating patients

Twelve participants (63%) from the intervention group provided free-text comments in response to the additional question included in the RAND-36 questionnaire. The main emerging themes were ‘finding it interesting and meaningful to track my physical activity’ and ‘hoping my participation helps others.’ In the control group, the primary themes identified from the responses of six participants (30%) were ‘the registration of daily PROMs was challenging when experiencing increased pain (“bad days”)’ and ‘hoping my participation helps others.

### Adverse events

No adverse events were reported or observed during the study.

### Overall assessment against pre-defined criteria of feasibility and acceptability

**Figure 3.**
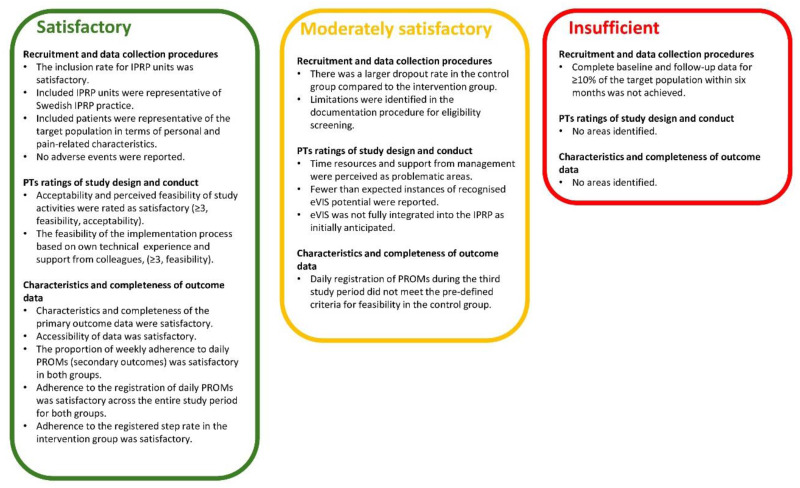
Overall assessment of satisfactory- (green), and moderately satisfactory- (yellow), and insufficient satisfactory (red) feasibility and acceptability in key areas of the assessment. PTs = physiotherapists.

The overall assessment of the R-RCT study design and conduct within IPRP suggests that the current study design and conduct are mainly feasible. However, a few areas were identified where feasibility and acceptability were assessed as moderately satisfactory, and one area rendered to have insufficient feasibility in comparison to the pre-defined criteria (Figure 3).

## Discussion

The results show mainly satisfactory feasibility and acceptability in recruitment and data collection procedures, in ratings from physiotherapists regarding trial design and conduct, and in characteristics and completeness of R-RCT outcome data. However, we identified potential threats to the feasibility of the study design and conduct. These threats include attrition bias, possible barriers for physiotherapists to accurately complete study activities, and decreased longitudinal adherence to daily PROMs in the control group – all risking to affect internal and external validity of the main trial.

### Strengths and limitations of the study

This pilot study involved extensive data collection from several data sources. Despite a study sample size on the smaller side of what is recommended,^63^ the sample was deemed satisfactory. It is important to note that this study was not designed to test the hypothesis, but rather to assess the feasibility and acceptability of the trial design and trial conduct of the R-RCT. While an intention-to-treat analysis will be conducted in the R-RCT to evaluate the intervention's effectiveness on both primary and secondary outcomes, a methodological decision was made in this pilot study to include only participants with complete baseline and follow-up data (i.e., a per protocol analysis) in the analyses. Consequently, any interpretation of the intervention's effect must consider the potential impact of this approach on the balance between groups. Furthermore, data collected in this study was derived from multiple sources, including outcome data from participating patients, data exported from the web application, and questionnaire data from physiotherapists, which formed the basis for the feasibility and acceptability assessments. In order to provide an overall assessment of the feasibility and acceptability of trial design and conduct, we applied, in accordance with recommendations for pilot studies,^40,41^ pre-defined feasibility criteria. However, such pre-defined criteria can potentially introduce researcher bias. To minimize such a risk, the applied criteria were chosen based on a literature review and were discussed within the author team. Furthermore, this pilot study included six small to medium-sized Swedish IPRP units. Although one of the unit's physiotherapists failed to provide ratings on trial design and trial conduct, the majority of units completed ratings which is considered a strength.

### Comparison of results to other studies

In this pilot study, the vast majority of the feasibility and acceptability aspects of trial design and conduct were assessed as “satisfactory”. The decision to assess the feasibility of recruitment and data collection procedures, physiotherapist ratings of feasibility and acceptability, the feasibility of outcome data's characteristics and completeness, and the occurrence of adverse events, was based on recommendations by Eldridge et al. (2016), Blatch-Jones et al. (2018), and Avery et al. (2016).^40–42^ The evaluation of the main trial's feasibility of recruitment and data collection procedures is well-recognised in randomised pilot studies,^64–67^ as satisfactory feasibility in these areas enhances the internal validity of the main trial. Despite moderately satisfactory feasibility in terms of patient inclusion time rate (which may be explained by the high impact that restrictions due to the Covid-19 pandemic had on the study), results showed notable differences in attrition rates between groups (15% in the intervention group and 37.5% in the control group). A difference in attrition was expected due to the study's six-month duration and that the control group participants were prompted to provide daily data without experiencing the potential benefits of the eVIS intervention. This result has also been seen in other randomised and non-randomised pilot trials involving participants with chronic diseases. Despite this, we determined that further refinement of the recruitment procedure was necessary. This includes providing more detailed information about the study's aim and design to eligible patients, particularly emphasising the important role that participants in the control group play, and increased number of digital reminders.^66,68,69^ Additionally, a review of the randomised groups revealed an imbalance in sex distribution (90% females in one group versus 60% in the other). Although the small sample, this will be monitored at additional time points throughout the data collection process in order to rule out randomisation failure.

Physiotherapists’ ratings on the feasibility and acceptability of trial design and conduct were collected, as successful implementation is highly dependent on the acceptability of the study design.^43,70^ The result yielded that physiotherapists assessed some areas as “moderately feasible”, resulting in minor revisions being made, primarily to the IPRP unit's information and education materials. In addition, results revealed that was various interpretations of how the eligibility screening procedures were supposed to be performed. Despite high ratings suggesting satisfactory eligibility of screening procedures, the later collected recruitment forms suggest that one unit solely registered patients eligible for the study, leaving information on non-eligible or not interested patients unknown. As this constitutes a clear risk of compromised external validity, measures were taken immediately to correct this. Despite limited data collection of patient feedback on what they perceived as relevant, the evaluated areas of feasibility and acceptability were limited to the perspectives of the researchers and the intervention deliverers (IPRP physiotherapists). In a systematic review, Bergevi et al. (2022) concluded that user perceptions of eHealth interventions promoting physical activity are of the utmost importance,^71^ and the lack of this perspective constitutes a limitation in current study. Physiotherapist ratings also revealed barriers to the study's conduct, such as time constraints and a lack of support from management. In response, we have begun exploring strategies to mitigate the impact of these barriers. These strategies include seeking support from administrative personnel at the IPRP unit and considering our participation in management meetings to emphasise the study's relevance.

Although participant adherence to providing secondary outcome data was mostly satisfactory in both groups, results identified the possible requirement of additional prompts in the control group to ensure daily registrations were made throughout the study period. Lastly, characteristics of the primary outcome in the R-RCT (physical health, PCS), confirms that the preliminary power calculation with MCID ≥3, was appropriate. One of the greatest strengths of this study was its pragmatic design, which entailed a conscious approach that did not interfere with how physiotherapists utilised eVIS within the setting, nor prompt participating patients to perform PROMs in an exaggerated manner. After assessing the results, we suspect that this may have rendered low ratings on the extent that the physiotherapists reported using eVIS as a supplementary measure in IPRP. This balance between steering how physiotherapists utilise the intervention compared to the pragmatic study design needs to be carefully considered moving forward.^43^

## Conclusion

In conclusion, the majority of the recruitment and data collection procedures, physiotherapist ratings, and the characteristics and completeness of outcome data were found to be feasible within a Swedish IPRP setting. However, there were a few exceptions, most of which were assessed as moderately satisfactory. For instance, the inclusion rate for IPRP units could be accelerated, and there was a notable difference in attrition rates between groups. This latter issue was addressed by providing specific study information to participants in the control group and increasing the frequency of digital reminders. Additionally, the need for enhanced instructions regarding the documentation of the eligibility screening process was identified, leading to revisions in both the educational material and written instructions.

Time constraints faced by physiotherapists during the implementation process were also identified as a potential risk to the performance of study activities. To mitigate this, measures such as providing support from administrative personnel at the unit may be beneficial. Furthermore, an increase in digital reminders is suggested to enhance the frequency of daily PROM registrations, particularly during the final third of the study period, despite overall data completeness being assessed as satisfactory.

The pre-defined criterion of complete baseline and follow-up data for 10% of the R-RCT goal population within the first six months was not fulfilled, resulting in an assessment of ‘insufficient’ feasibility and acceptability. This shortfall may be attributed to the limited number of IPRP units initially recruiting participants, as well as decreased patient throughput due to the pandemic. Overall, the feasibility and acceptability of the R-RCT study design and conduct were generally affirmed, with a handful of identified threats effectively remedied.

## Supplemental Material

sj-docx-1-dhj-10.1177_20552076241299648 - Supplemental material for Feasibility and acceptability of design and conduct of a registry-based randomised clinical trial evaluating eVIS as a digital support for physical activity in interdisciplinary pain rehabilitation programs: A randomised pilot study Supplemental material, sj-docx-1-dhj-10.1177_20552076241299648 for Feasibility and acceptability of design and conduct of a registry-based randomised clinical trial evaluating eVIS as a digital support for physical activity in interdisciplinary pain rehabilitation programs: A randomised pilot study by Veronica Sjöberg, Andreas Monnier, Elena Tseli, Riccardo LoMartire, Maria Hagströmer, Mathilda Björk, Björn Äng and Linda Vixner in DIGITAL HEALTH
