# Supplementary material for: Feasibility and acceptability of design and conduct of a registry-based randomised clinical trial evaluating eVIS as a digital support for physical activity in interdisciplinary pain rehabilitation programs: A randomised pilot study
Source: Digit Health. 2024 Nov 25;10:20552076241299648. doi: 10.1177/20552076241299648 (PMC11590142; doi:10.1177/20552076241299648)
Supplement: sj-docx-1-dhj-10.1177_20552076241299648 - Supplemental material for Feasibility and acceptability of design and conduct of a registry-based randomised clinical trial evaluating eVIS as a digital support for physical activity in interdisciplinary pain rehabilitation programs: A randomised pilot study [file sj-docx-1-dhj-10.1177_20552076241299648.docx]

CONSORT checklist of information to include when reporting a pilot trial

| **Section/topic and item no** | **Extension for pilot trials** | **Page No where item is reported** |
| --- | --- | --- |
| Title and abstract | | |
| 1 a | Identification as a pilot or feasibility randomised trial in the title | 1 |
| 1 b | Structured summary of pilot trial design, methods, results, and conclusions | 2 |
| Introduction | | |
| Background and objectives | | |
| 2 a | Scientific background and explanation of rationale for future definitive trial, and reasons for randomised pilot trial | 3, 4 |
| 2 b | Specific objectives or research questions for pilot trial | 2, 4 |
| Methods | | |
| 3 a | Description of pilot trial design (such as parallel, factorial) including allocation ratio | 5 |
| 3 b | Important changes to methods after pilot trial commencement (such as eligibility criteria), with reasons | Only small changes in written and oral information material have been made |
| Participants | | |
| 4 a | Eligibility criteria for participants | 6-7 |
| 4 b | Settings and locations where the data were collected | 5 |
| 4 c | How participants were identified and consented | 7 |
| Interventions | | |
| 5 | The interventions for each group with sufficient details to allow replication, including how and when they were actually administered | 6 |
| Outcomes | | |
| 6a | Completely defined prespecified assessments or measurements to address each pilot trial objective specified in 2 b including how and when they were assessed | 8-9 |
| 6 b | Any changes to pilot trial assessments or measurements after the pilot trial commenced, with reasons | No changes to assessment or measurement have been made |
| 6 c | If applicable, prespecified criteria used to judge whether, or how, to proceed with future definitive trial | 9-11 |
| 7 a | Rationale for numbers in the pilot trial | 20 |
| 7 b | When applicable, explanation of any interim analyses and stopping guidelines | NA |
| Randomisation  Sequence generation | | |
| 8 a | Method used to generate the random allocation sequence | 5 |
| 8 b | Type of randomization, details of any restriction (such as blocking and block size) | 5 |
| Allocation concealment mechanism | | |
| 9 | Mechanism used to implement the random allocation sequence (such as sequentially numbered containers), describing any steps taken to conceal the sequence until interventions were assigned | 5 |
| Implementation | | |
| 10 | Who generated the random allocation sequence, enrolled participants, and assigned participants to intervention | 5 |
| Blinding | | |
| 11 a | If done, who was blinded after assignment to interventions (eg. Participants, care providers, those assessing outcomes), and how | Blinding after allocation was not possible due to the nature of the intervention |
| 11 b | If relevant, description of the similarity of the interventions | Not relevant |
| Analytical methods | | |
| 12 a | Methods used to address each pilot trial objective whether qualitative or quantitative | 6-11 |
| Results | | |
| 13 a | For each group, the numbers of participants who approached and/or assessed for eligibility, randomly assigned, received intended treatment and were assessed for each objective | 11-12 |
| Recruitment | | |
| 14 a | Dates defining the periods of recruitment and follow-up | 12 |
| 14 b | Why the pilot trial ended or was stopped | 7 |
| Baseline data | | |
| 15 | A table showing baseline demographic and clinical characteristics for each group | 12, 13 |
| Numbers analysed | For each objective, number of participants (denominator) included in each analysis. If relevant, these numbers should be by randomised group | 13 |
| Outcomes and estimation | | |
| 17 a | For each objective, results including expressions of uncertainty (such as 95% confidence interval, CI) for any estimates. If relevant, these results should be by randomised group | 11-19 (expression of 95% CI for percentage estimates of improved, unchanged, deteriorated in the R-RCT´s primary outcome) |
| Ancillary analyses | | |
| 18 | Results of any other analyses performed that could be used to inform the future definitive trial | Not relevant |
| Harms | | |
| 19 | All important harms or unintended effects in each group | 19 |
| 19 a | If relevant, other important unintended consequences | Not relevant |
| Discussion | | |
| 20 Limitations | Pilot trial limitations, addressing sources of potential bias and remaining uncertainty about feasibility | 20-22 |
| 21 Generalisability | Generalisability (applicability) of pilot trial methods and findings to future definitive trial and other studies | 20-22 |
| 22 Interpretation | Interpretation consistent with pilot trial objectives and findings, balancing potential benefits and harms, and considering other relevant evidence | 20-22 |
| 22 a | Implications for progression from pilot to future definitive trial, including any proposed amendments | 22 |
| Registration | | |
| 23 | Registration number for pilot trial and name of trial registry | 5 |
| Protocol | | |
| 24 | Where the pilot trial protocol can be accessed, if available | 23 |
| Funding | | |
| 25 | Sources of funding and other support (such as supply of drugs), role of funders | 23 |
| 26 | Ethical approval or approval by research committee, confirmed with reference number | 23 |
